# Construction and validation of a histone acetylation-related lncRNA prognosis signature for ovarian cancer

**DOI:** 10.3389/fgene.2022.934246

**Published:** 2022-10-12

**Authors:** Xiao-Qian Hu, Xiao-Chong Zhang, Shao-Teng Li, Tian Hua

**Affiliations:** ^1^ Department of Oncology, Affiliated Xingtai People Hospital of Hebei Medical University, Xingtai, China; ^2^ Department of Gynecology, Affiliated Xingtai People Hospital of Hebei Medical University, Xingtai, China

**Keywords:** ovarian cancer, long non-coding RNA, cluster, histone acetylation, bioinformatics

## Abstract

Ovarian cancer (OC) leads to the most deaths among gynecological malignancies. The various epigenetic regulatory mechanisms of histone acetylation in cancer have attracted increasing attention from scientists. Long non-coding RNA (lncRNA) also plays an important role in multiple biology processes linked to OC. This study aimed to identify the histone acetylation-related lncRNAs (HARlncRNAs) with respect to the prognosis in OC. We obtained the transcriptome data from Genotype-Tissue Expression (GTEx) project and The Cancer Genome Atlas (TCGA); HARlncRNAs were first identified by co-expression and differential expression analyses, and then univariate Cox regression and the least absolute shrinkage and selection operator (LASSO) were used to construct the HARlncRNAs risk signature. Kaplan–Meier analysis, time-dependent receiver operating characteristics (ROC), univariate Cox regression, multivariate Cox regression, nomogram, and calibration were conducted to verify and evaluate the risk signature. Gene set enrichment analysis (GSEA) in risk groups were conducted to explore the tightly correlated pathways with the risk group. A risk signature with 14 HARlncRNAs in OC was finally established and further validated in the International Cancer Genome Consortium (ICGC) cohort; the 1-, 3-, and 5-year ROC value, nomogram, and calibration results confirmed the good prediction power of this model. The patients were grouped into high- and low-risk subgroups according to the risk score by the median value. The low-risk group patients exhibited a higher homologous recombination deficiency (HRD) score, LOH_frac_altered, and mutLoad_nonsilent. Furthermore, consensus clustering analysis was employed to divide OC patients into three clusters based on the expression of the 14 HARlncRNAs, which presented different survival probabilities. Principal component analysis (PCA) and t-distributed stochastic neighbor embedding (t-SNE) were also performed to evaluate the three clusters. In conclusion, the risk signature composed of 14 HARlncRNAs might function as biomarkers and prognostic indicators with respect to predicting the response to the anti-cancer drugs in OC.

## Introduction

Globally, ovarian cancer (OC) is the first cause of death within all gynecological malignancies, leading to approximately 185,000 deaths each year (Miller et al., 2020). The 5-year survival rate of advanced stage OC is around 30%, with most deaths occurring within 24 months of diagnosis (Ataseven et al., 2016). Due to the complexity of clinical symptoms and biological and molecular features, OC is considered to be one of the most difficult tumors to overcome. The standard first-line chemotherapy for those with Stage Ic disease or higher is a combination treatment of platinum agents with paclitaxel after primary cytoreductive surgery (Kim et al., 2012). However, either primary or acquired resistance to platinum compounds is the main obstacle to successful treatment. Unfortunately, the biomarkers that could be used as an exact index for diagnosis, prognosis prediction, and monitoring of treatment for OC patients are yet to be found.

Compelling pieces of evidence have confirmed epigenetics, an inheritable phenomenon, made immeasurable contributions to the initialization and development of tumors (Yang et al., 2018; Conteduca et al., 2021). It mainly regulates gene expression through histone modification, DNA methylation, non-coding RNA regulation, and chromatin structure rebuilding (Li, 2021). Histone acetylation, a critical form of histone modification, may change chromatin architecture and regulate gene expression *via* opening or closing the chromatin structure (Martin et al., 2021). In addition to regulating gene expression, histone acetylation has been found to affect multiple cellular processes, such as angiogenesis regulation, cell cycle, DNA repair, DNA stress response, apoptosis, and autophagy (Liu et al., 2019). The noticeable features of histone acetylation are dynamic and reversible which are regulated by histone acetyltransferases (HATs) and histone deacetylases (HDACs) (Hai et al., 2021). Previous research studies have reported that aberrant expression of HATs and HDACs were associated with cancer pathologies (Mi et al., 2017). HDACs were observed to be overexpressed in different human tumor cell lines, identified as key targets of tumorigenesis (Rajan et al., 2020). Furthermore, an imbalance between HATs and HDACs was confirmed to be linked with the pathogenesis of OC (Huang et al., 2016). In addition, histone deacetylase inhibitors (HDACis), as a class of epigenetic regulatory drugs, have attracted increasing attention due to their important role in regulating cell cycle, proliferation, differentiation, and activity. Many HDACis have entered preclinical and clinical trials in OC with a tempting potential of anti-cancer ability, such as Trichostatin A (Muscolini et al., 2008) and Belinostat (Dizon et al., 2012). Although the mechanism of how histone acetylase changes in OC remains unclear presently, abnormal histone acetylase occurs frequently in ovarian malignant tumors and is considered to contribute to the initiation and development of OC (Xie et al., 2021). Hence, the exploration of histone acetylase has very high expectations for providing attractive biomarkers for diagnosis, prognosis, and therapeutic targets in women OC. Recently, Dai and Ye established a histone acetylation-based gene signature with a good predictive effect on the prognosis of OC (Dai and Ye, 2022). It was universally known that the initiation and progression of OC are intimately connected not only with the abnormal expression of protein-coding mRNAs but also with the non-coding RNAs. It was worth mentioning the remarkable role of long non-coding RNAs (lncRNAs) in multiple biological processes related to cancers. lncRNAs promote energy metabolism and cancer progression through posttranslational modifications of key metabolism-related proteins, containing ubiquitination, phosphorylation, and acetylation (Hu et al., 2017; Tan et al., 2021). To make an all-around understanding of the regulation network of histone acetylation in OC, it was necessary to explore the possible functions of the histone acetylation-related lncRNAs (HARlncRNAs), which might provide more information to aid the regulation of histone acetylation in OC.

In this present study, we aimed to build a HARlncRNA signature associated with OC patients’ clinical outcomes. The risk score was calculated according to the HARlncRNA signature. The sensitivity and specificity of the HARlncRNA signature were evaluated by time-dependent receiver operating characteristic (ROC) analysis. Some analyses were investigated based on the risk scores, such as nomogram, calibration, Kaplan–Meier analysis, GSEA, and the correlation with the HRD score. Furthermore, consensus clustering analysis was used to identify OC subtypes based on the HARlncRNAs.

## Materials and methods

### Data extraction

The RNA-seq omics data and corresponding clinical information of 375 OC patients were downloaded from the UCSC Xena database (https://xenabrowser.net/) (Goldman et al., 2020), and 88 normal human ovarian samples RNA-seq omics data were also downloaded from the UCSC Xena database related to the Genotype-Tissue Expression (GTEx, https://xenabrowser.net). The RNA-seq omics data and corresponding clinical information of 93 OC patients from the International Cancer Genome Consortium (ICGC) were downloaded from https://dcc.icgc.org/ (Hudson et al., 2010).

### Construction of the prognostic-related predictive signature

A total of 36 histone acetylation-related genes (HARGs) were obtained by reference to previous reports about histone acetylase (Xu et al., 2022), and then all lncRNAs were filtered out through a biotype in file GRCh38.p13 gtf. To discern HARlncRNAs, we applied the Pearson correlation to appraise the relevance between HARGs and lncRNAs and we chose 1,201 HARlncRNAs by the standards of correlation coefficient > 0.4 and *p*-value < 0.001; then, we conducted the Wilcoxon test to filter 628 differentially expressed lncRNAs between samples of TCGA and GTEx database in specific criteria (|log2FC| > 1 and FDR <0.05). The entire dataset was split into train and test datasets at a proportion of 1:1. In total, 27 HARlncRNAs were obtained after univariate Cox regression analysis was performed in the TCGA cohort (R package “survival”); afterward, we used the least absolute shrinkage, selection operator (LASSO), Cox regression model, and multivariate stepwise Cox regression (R package “glmnet”, “survival”), and we got 14 HARlncRNAs in the risk signature ultimately. The 375 OC patients were grouped into two groups (low- and high-risk) on account of the median value. The ROC curve was plotted to estimate the predictive value of the prognostic gene signature for overall survival (OS) (R package “timeROC”).

### Nomogram and calibration

We used the multivariate Cox regression analysis of clinical information and risk score to build the nomogram (R package “rms”); the calibration curves of 1 , 3 , and 5 years were used to examine the correctness of the nomogram.

### Gene set enrichment analysis (GSEA)

GSEA v4.2.3 from the MSigDB database (http://software.broadinstitute.org/gsea/msigdb/) (Subramanian et al., 2005) was conducted to find the closely related GO function between low- and high-risk groups; the criterion of selection was FDR q-value < 0.25, nominal *p*-value < 0.05, and |NES| ≥ 1.5.

### TCGA DNA damage repair (DDR) data resources

The HRD score was retrieved from a previous study (Knijnenburg et al., 2018). The reverse phase protein array (RPPA) data were retrieved from a previous study (Akbani et al., 2014). eCARD (expression CDF transformation of rank distribution) was retrieved from a previous study (Zimmermann et al., 2016).

### Determination of molecular subtypes in ovarian cancer

ConsensusClusterPlus extends the consensus clustering algorithm (including item tracking, item-consensus, and cluster-consensus plots), and we conducted a consensus matrix and CDF plot to determine the supreme cluster number of subtypes (Wilkerson and Hayes, 2010) and found three clusters. t-distributed stochastic neighbor embedding (t-SNE) and principal component analysis (PCA) were performed by the Rtsne R package.

### Statistical analysis

All statistical analyses were done using R software 4.1.1. *p*-value < 0.05 was considered statistically significant unless noted otherwise. The log-rank test was used to compute the log-rank *p*-value and hazard ratio (HR), the Wilcoxon test was used for comparisons between two groups, and the Kruskal–Wallis test was used for more than two groups.

## Results

**FIGURE 1:**
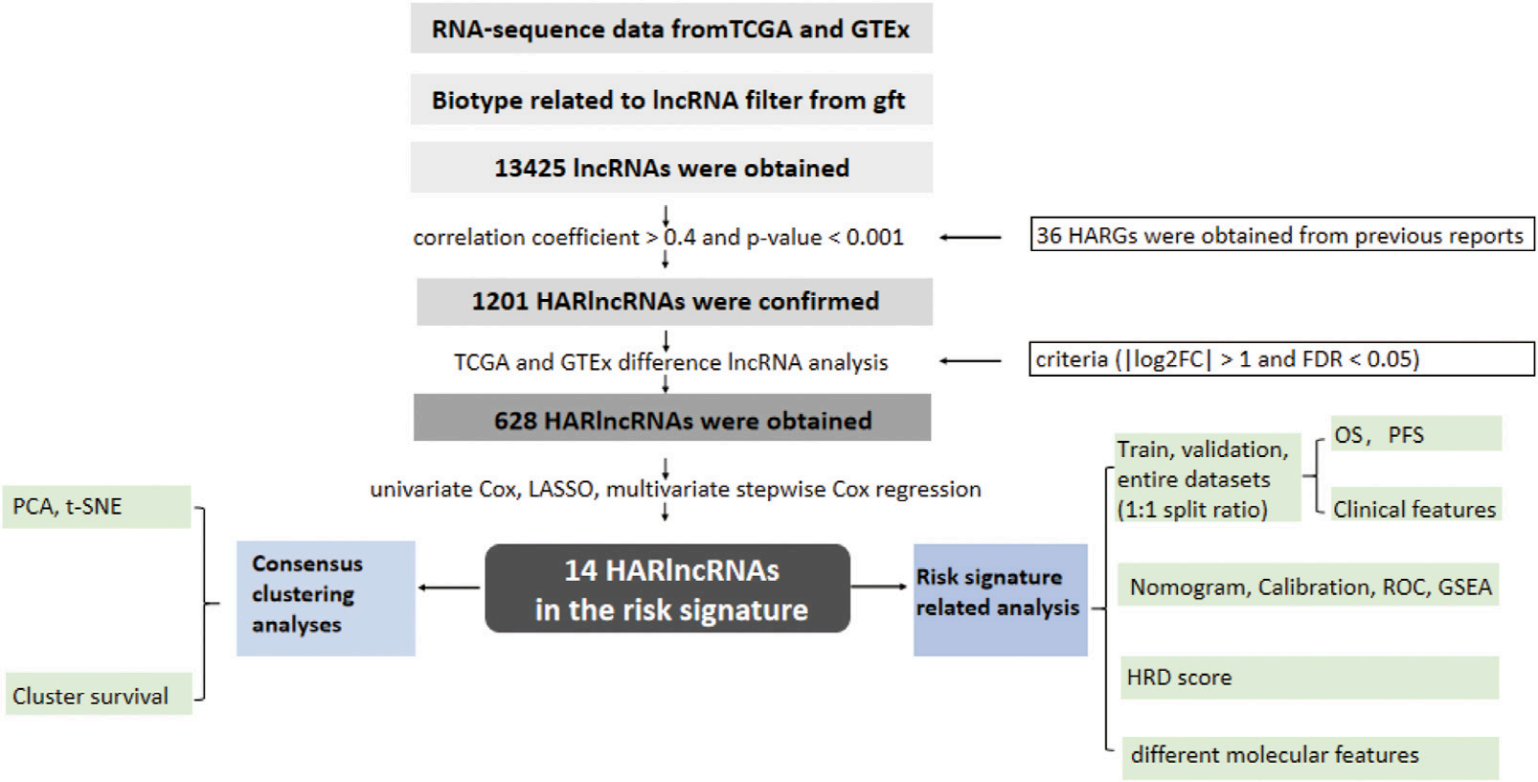
Flow diagram of the study.

In this study, the data of 375 OC and 88 normal patient samples from the TCGA and GTEx were collected. The data of 93 OC subjects were collected from ICGC. The workflow diagram of the study is presented in Figure 1.

### Establishment of HARlncRNAs in ovarian cancer

**FIGURE 2:**
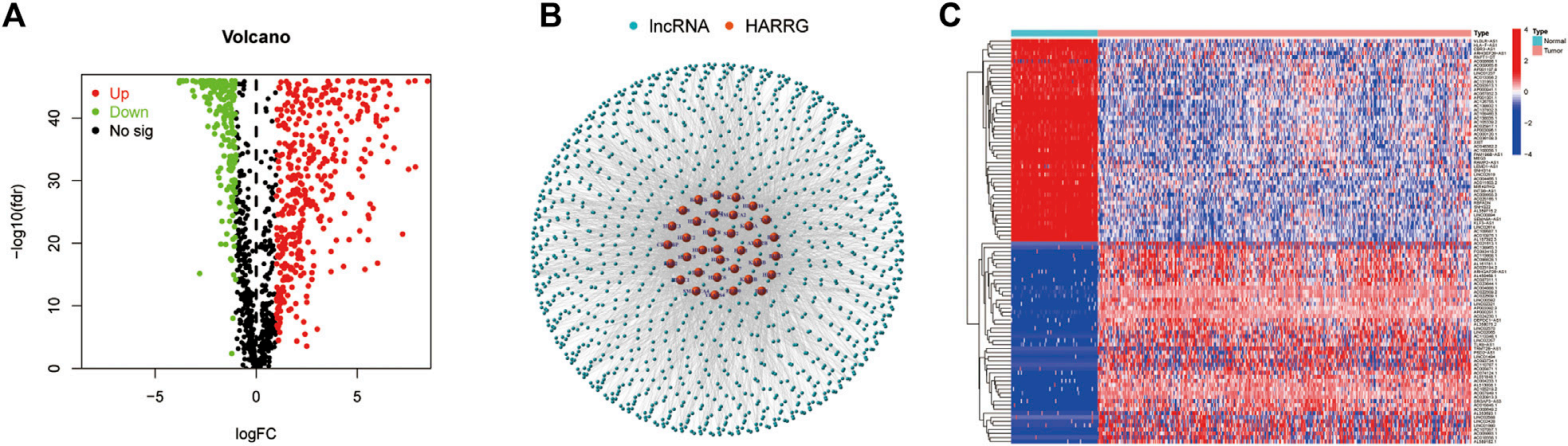
Identification of histone acetylation-related lncRNAs (HARlncRNAs) in patients with ovarian cancer (OC). **(A)** Volcano plot of the differentially expressed HARlncRNAs. Green points indicate the significantly downregulated genes. Red points indicate the significantly upregulated genes. **(B)** Networks between histone acetylation-related genes and lncRNAs (correlation coefficients >0.4 and *p* < 0.001). **(C)** Top 50 differentially expressed HARlncRNAs which were sorted by Log2 fold change between tumor and normal patients.

A total of 36 HARGs were established from the previous reports about histone acetylation. In total, 1,202 HARlncRNAs were obtained on the foundation of correlation coefficients of 36 HARGs and 13,425 lncRNAs (correlation coefficients >0.4 and *p* < 0.001). Then, 628 differentially expressed lncRNAs (|Log2FC| > 1 and *p* < 0.05) between OC tumor and normal patient samples were ultimately confirmed. Compared to the normal tissues, 419 HARlncRNAs were upregulated, and the other 209 were downregulated in ovarian cancer (OC) samples (Figure 2A). The network figure and data between HARGs and lncRNAs were shown in Figure 2B and Supplementary Table S1. The top 50 differentially expressed HARlncRNAs, which were sorted by Log2 fold change between tumor and normal patient samples, were visualized in Figure 2C.

### Signature construction and validation of HARlncRNAs in ovarian cancer

**FIGURE 3:**
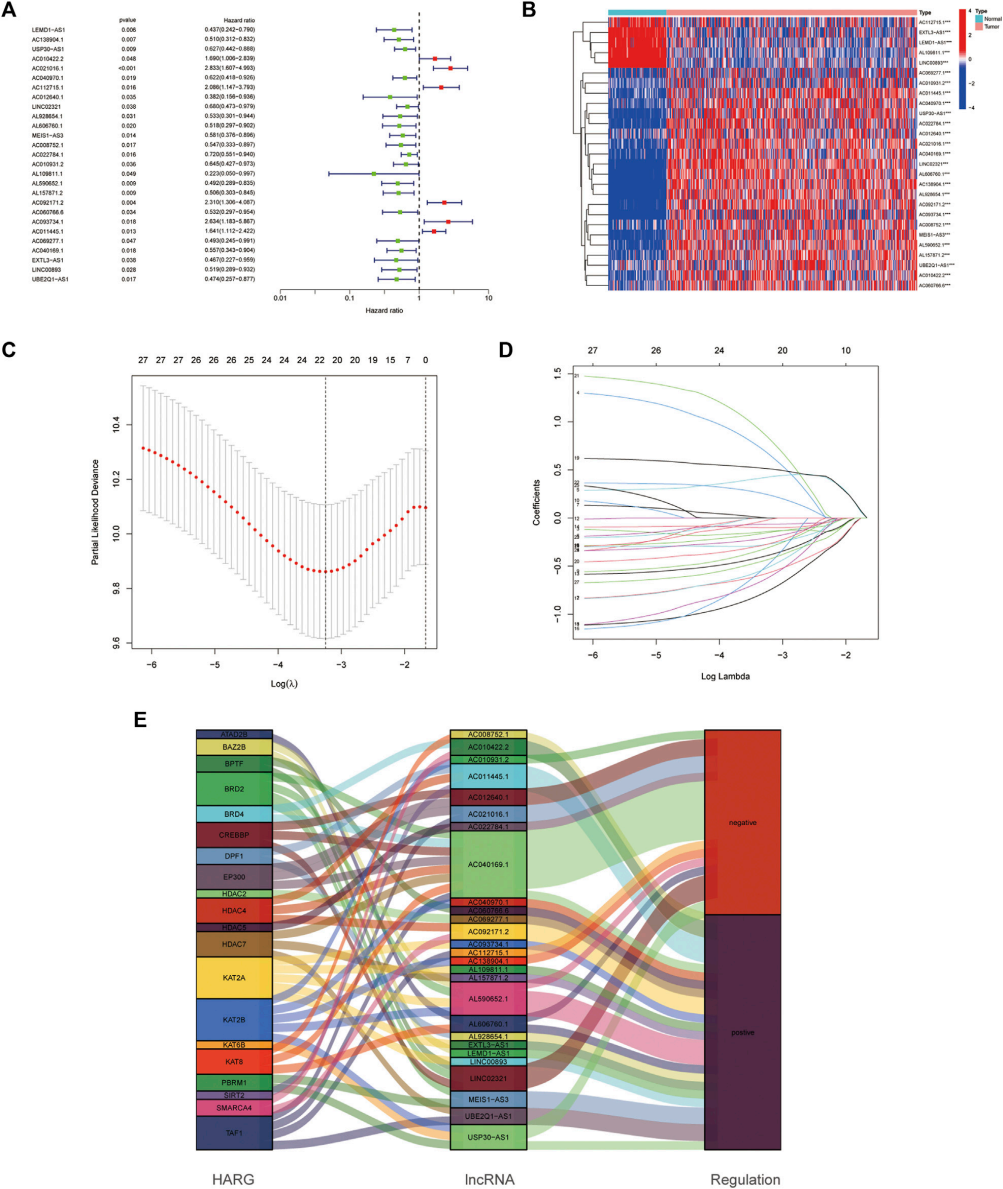
Establishment of the histone acetylation-related lncRNAs (HARlncRNAs) prognostic model in ovarian cancer (OC). **(A)** Prognostic-associated lncRNAs were extracted using univariate Cox regression analysis. **(B)** Expression profiles of 27 prognostic HARlncRNAs. **(C)** 10-fold cross-validation for variable selection in the LASSO regression analysis. **(D)** LASSO coefficient profile of HARlncRNAs. **(E)** Sankey diagram of pyroptosis genes and HARlncRNAs.

Among the 628 HARlncRNAs, 27 HARlncRNAs were found to be significantly correlated with the overall survival (OS) of OC patients according to univariate Cox regression analysis (*p* < 0.05, Figure 3A). The heatmap of the 27 HARlncRNAs was shown in Figure 3B. To avoid overfitting the prognostic signature, we performed the LASSO regression on the 27 HARlncRNAs; furthermore, we conducted multivariant Cox regression of the 27 HARlncRNAs. Of them, 14 HARlncRNAs were exacted to be related with histone acetylation in OC when the first-rank value of Log(λ) was the minimum likelihood of deviance (Figures 3C,D). Furthermore, we plotted the regulation between the 27 HARlncRNAs and HARGs in the Sankey diagram, and most relationships between HARGs and HARlncRNAs were positive (Figure 3E).

Afterward, we computed the risk score as follows:

LEMD1−AS1×(−1.0520)+AC138904.1×(−0.8239)+AC010422.2×(1.3250)+AC021016.1×(0.5876)+LINC02321×(−0.6593)+AC008752.1×(−0.7215)+AL590652.1×(−0.8644)+AL157871.2×(−1.0107)+AC092171.2×(0.4927)+AC060766.6×(−0.6976)+AC093734.1×(1.4787)+AC011445.1×(0.5794)

AC040169.1×(−0.4930)+UBE2Q1−AS1×(−0.6988).

**FIGURE 4:**
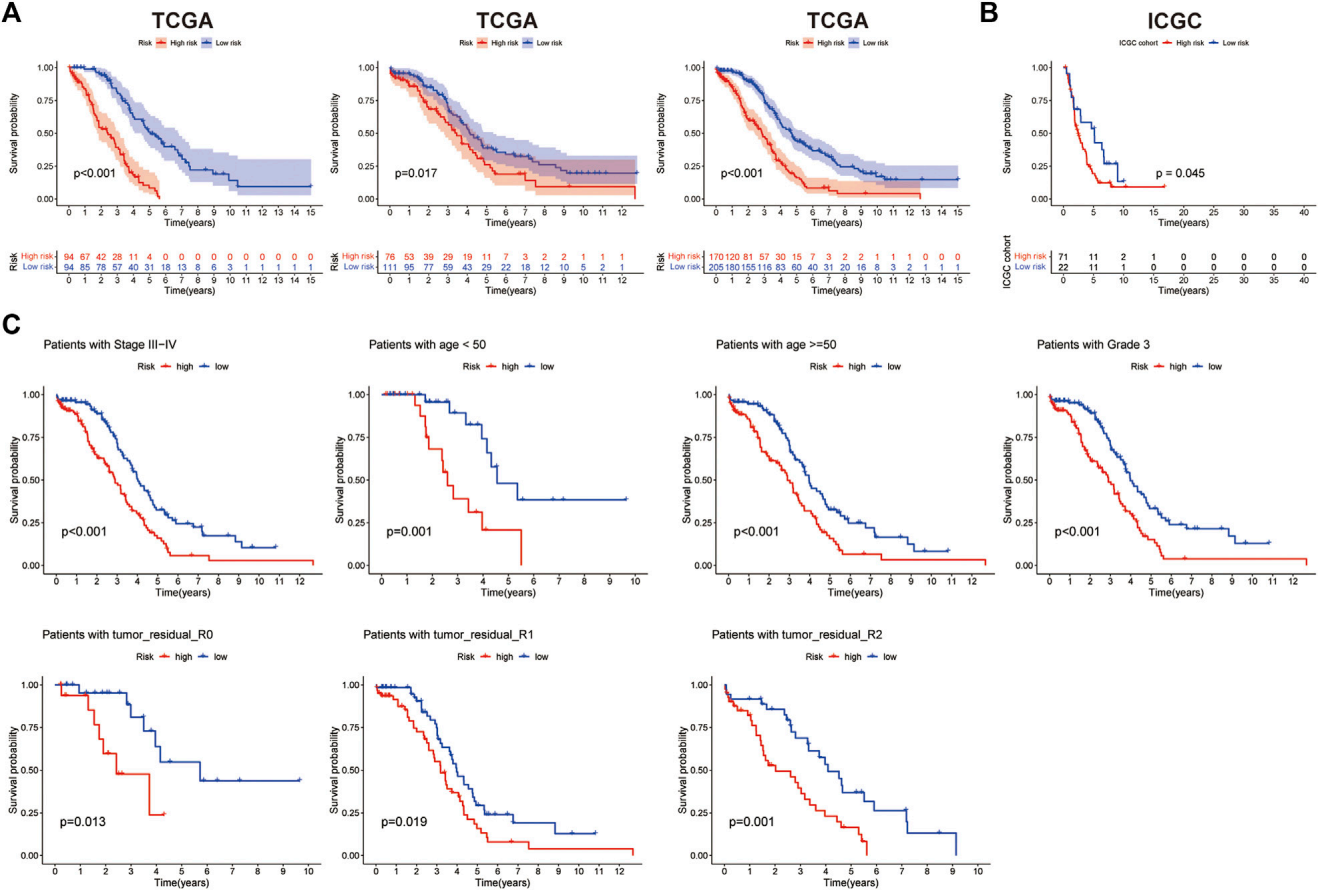
Prognosis value of the 14-histone acetylation-related lncRNA (HARlncRNAs) signature in the train, test, and all sets. **(A)** Kaplan–Meier survival curves of overall survival (OS) of patients between high- and low-risk subgroups in the train, test, and all sets, respectively. **(B)** Kaplan–Meier survival curves of OS of patients between high- and low-risk subgroups in the ICGC cohort. **(C)** Kaplan–Meier survival curves of OS prognostic value stratified by age, grade, stage, and tumor residual size between high- and low-risk subgroups in the entire set.

The risk score median value was used as a cutoff to divide 375 OC cases into the high- and low-risk groups. The distribution of the risk score was plotted in the train, test, and entire dataset, respectively, which all demonstrated that patients in the high-risk group had poorer OS than those patients in the low-risk group (Figure 4A). In addition, the result from the ICGC cohort was consistent with that from the TCGA cohort: the shorter OS was shown in the high-risk group patients (Figure 4B). Kaplan–Meier analysis was performed after risk stratification using FIGO stage, age, grade, and tumor residual size (Figure 4C). Patients in the low-risk group showed improved OS compared with patients with high-risk for stage III–IV (*p* < 0.001), age<50 (*p* < 0.001), age≥50 (*p* < 0.001), grade 3 (*p* < 0.001), and tumor residual size (*p* < 0.001).

### Estimation and evaluation of nomogram

**FIGURE 5:**
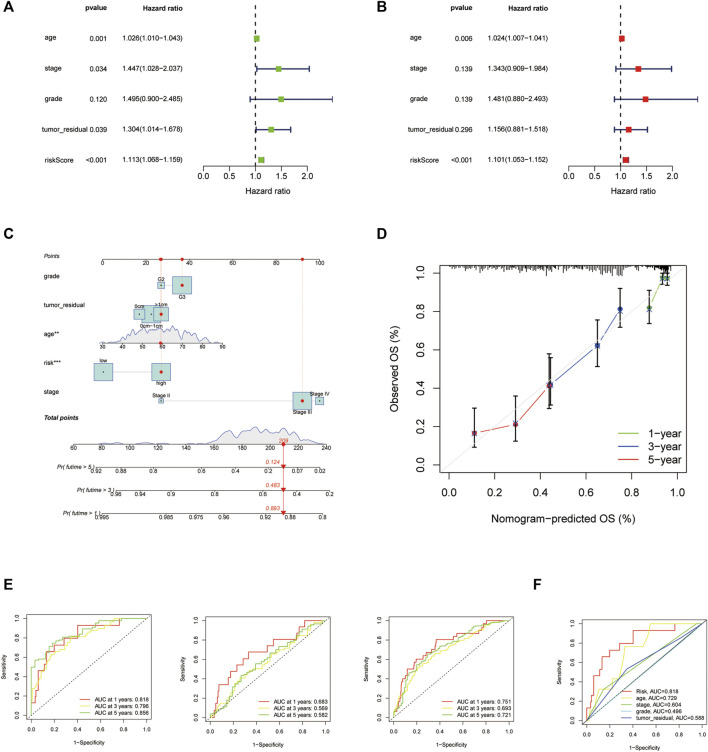
Nomogram and assessment of the risk signature based on 14 histone acetylation-related lncRNAs (HARlncRNAs). **(A,B)** Univariate Cox and multivariate regression analyses of the risk score and clinical factors with overall survival (OS). **(C)** Nomogram that integrated the risk score, age, grade, stage, and tumor residual size predicted the probability of the 1-, 3-, and 5-year OS. **(D)** Calibration curves for the 1-, 3-, and 5-year OS. **(E)** The 1-, 3-, and 5-year time-dependent receiver operating characteristic (ROC) curves of the train, test, and all sets, respectively. **(F)** ROC curves of the risk score, nomogram total score, and clinical characteristics.

A nomogram was built to forecast the survival risk in OC patients on the basis of the total TCGA cohort. First, the univariate and multivariate Cox regression analyses were used to determine the independent prognostic values of the risk score from the 14 HARlncRNAs, age, FIGO stage, histological grade, and tumor residual size in OC. The results were shown in Figures 5A,B, demonstrating that the risk score of this model was an independent prognostic factor for OC patients (*p* < 0.001, HR = 1.113, 95% CI = 1.068–1.159, Figure 5A; *p* < 0.001, HR = 1.101, 95% CI = 1.053–1.152, Figure 5B). We also figured out another independent prognostic factor: age (*p* = 0.006, HR = 1.024, 95% CI = 1.007–1.041, Figure 5B). Moreover, a nomogram was conducted for predicting the 1-, 3-, and 5-year OS incidences of OC patients (Figure 5C). We also used the 1-, 3-, and 5-year calibration plots to prove that the nomogram had a good concordance with the prediction of 1-, 3-, and 5-year OS (Figure 5D). In order to assess the sensitivity and specificity of the risk model on the prognosis, the ROC was performed. We also illustrated the outcomes of ROC with the area under the ROC curve (AUC). The HARlncRNAs risk model displayed AUC values of 0.818, 0.796, and 0.856 at 1, 3, and 5 years in the ROC analysis in the train set, respectively. The AUC values in the test set were 0.683, 0.569, and 0.582 at 1, 3, and 5 years. In the entire set, the AUC values were 0.751, 0.693, and 0.721 at 1, 3, and 5 years (Figure 5E), revealing effective predictions of survival by the HARlncRNAs risk signature. Furthermore, compared with other clinical characteristics including age (AUC = 0.729), FIGO stage (AUC = 0.604), histologic grade (AUC = 0.496), and tumor residual size (AUC = 0.588), there was a higher AUC value 0.818 at a 1-year OS time for the risk score (Figure 5F). These data suggested that the risk score model might possess higher sensitivity and accuracy in predicting the prognosis of patients with OC.

### Functional analysis of the 14 HARlncRNAs signature

**FIGURE 6:**
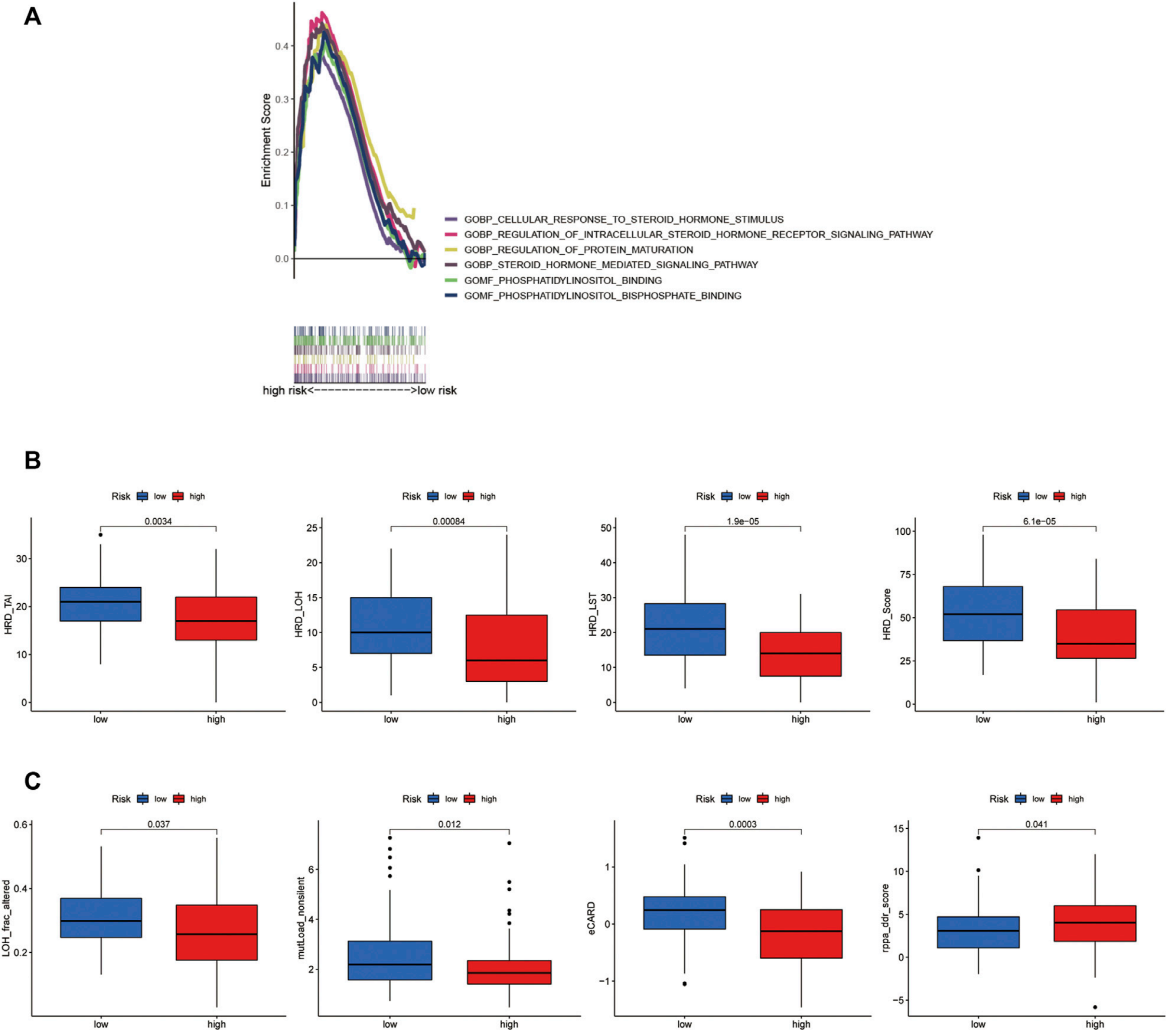
Different molecular characteristics between the groups. **(A)** GSEA of the significant GO terms significantly enriched in the high-risk group. (*p* < 0.05; FDR <0.25; |NES| > 1.5). **(B)** Comparison of the homologous recombination deficiency (HRD) status between the high- and low-risk groups (*p* < 0.05). **(C)** Comparisons of some other molecular features between the risk groups (*p* < 0.05).

For the investigation of differences in biological functions between high- and low-risk groups on the basis of the risk score, we used GSEA software to search for the GO terms in the entire set. The related GO terms in the low-risk group were GOBP cellular response to steroid and regulation of protein maturation. GOMF phosphatidylinositol binding and phosphatidylinositol bisphosphate binding (Figure 6A).

### Different molecular characteristics between the high- and low-risk groups

Genomic scarring with large-scale genome instability has been attributed to HRD (Watkins et al., 2014). We calculated an HRD score, combined from HRD-LOH (Abkevich et al., 2012), LST (large-scale state transitions) (Popova et al., 2012), and TAI (number of telomeric allelic imbalances) scores (Birkbak et al., 2012). The score of HRD, HRD_LOH, HRD_ LST, and HRD_TAI was confirmed to be higher in the low-risk group than that in the high-risk group (*p* < 0.001, Figure 6B). At the same time, for some new molecular indicators, the low-risk group owned the higher level of LOH_frac_altered and mutLoad_nonsilent (*p* = 0.037, *p* = 0.012, Figure 6C). In addition, the higher level of eCARD score was observed in the low-risk group (*p* < 0.001, Figure 6C), which exhibited a consistent association with OS among high-grade ovarian cancer patients in recent research studies (Zimmermann et al., 2016).

### Identification of the three distinct HARlncRNAs expression subtypes in ovarian cancer

**FIGURE 7:**
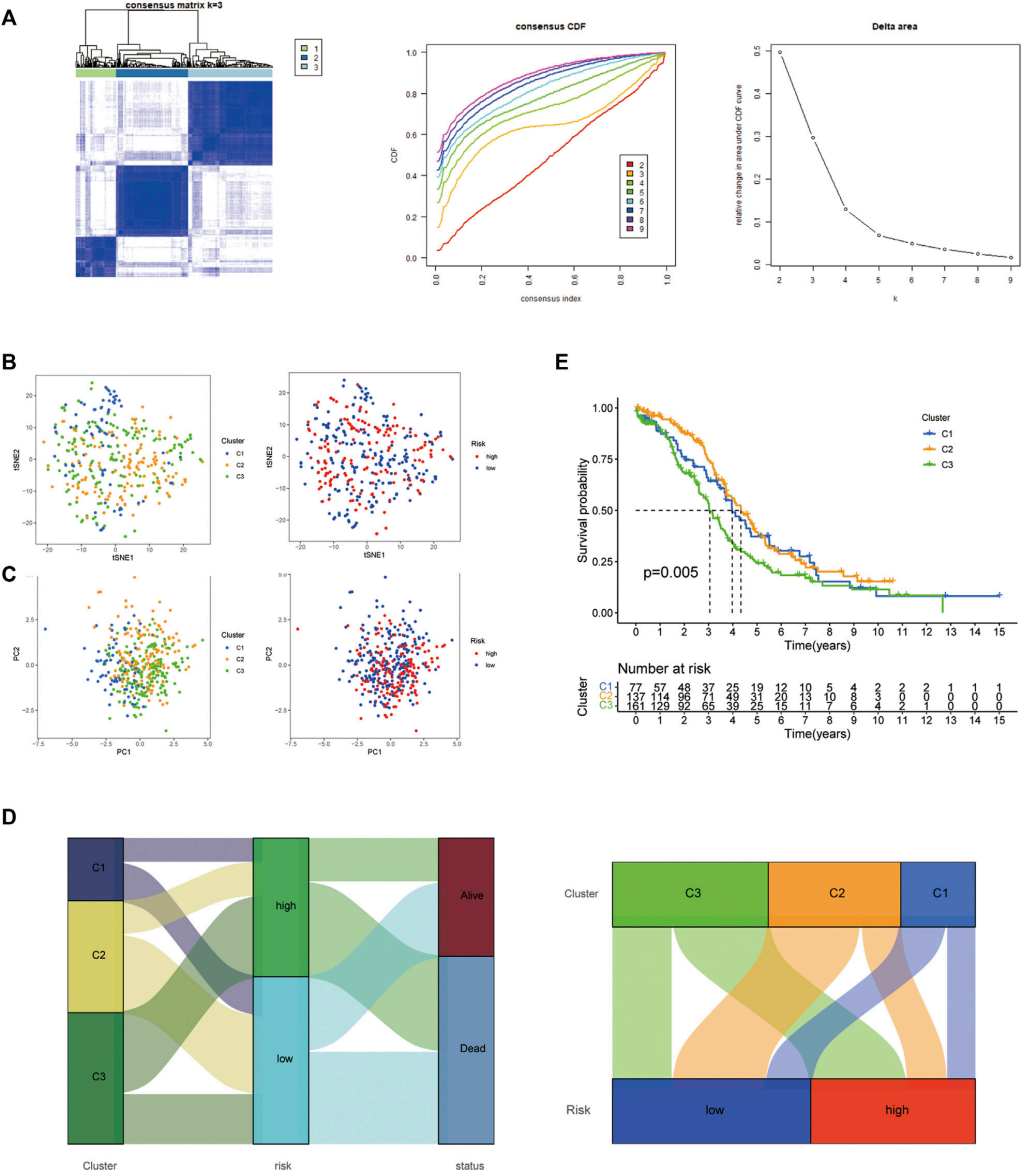
Three distinct expression clusters characterized by consensus clustering analysis. **(A)** Patients are divided into three clusters by ConsensusClusterPlus. **(B)** t-SNE of risk groups and clusters. **(C)** Principal component analysis (PCA) of risk groups and clusters. **(D)** Sankey diagram of clusters with their risk types and survival status. **(E)** Kaplan–Meier survival curves of overall survival (OS) in three clusters.

We used consensus clustering on the basis of the 14 HARlncRNAs expression which came from the risk model, three distinct clusters were displayed (Figure 7A); then, t-SNE and PCA analysis of 14 HARlncRNAs expression were clearly divided into three clusters, and the pre-defined high- and low-risk groups could also be divided into two clusters (Figures 7B,C), and the Sankey diagram was adopted to display relationships of clusters with their risk types, clusters, and survival status (Figure 7D). Survival analysis demonstrated a significant difference between the three clusters. Cluster 3 seemed to show the lowest survival probability (Figure 7E).

## Discussion

OC is one of the most common causes of mortality among gynecologic cancers in the world, of which OC accounts for the greater proportion. One of the reasons is the lack of effective biomarkers or risk models to guide treatments. In recent years, lncRNAs have attracted a lot of attention due to its broad spectrum of the content of biological effects in oncogenesis and tumor progression; with the development of diverse potential drugs targeting epigenetic regulators in preclinical and clinical trials (Cheng et al., 2019), the role of histone acetylation in cancer biological processes have become more and more important. In this study, we constructed a novel risk signature to predict prognosis and the survival for OC based on the histone acetylation-associated lncRNAs, and validated it in another ICGC cohort; the prediction efficiency was verified by ROC, nomogram, and a calibration curve. The response to target treatment (PARP inhibitors, PARPis) might be different between low- and high-risk groups due to significantly different molecular characteristics. In addition, the 375 OC patients were divided into three clusters based on the expression of the 14 HARlncRNAs by consensus clustering analysis, presenting the patients in different clusters that presented different survival probabilities.

LncRNA-mediated gene expression regulation might involve epigenetic mechanisms through direct interaction with proteins with epigenetic mechanisms, including histone modification and chromatin remodeling (Zhang et al., 2020). The interaction between differentially expressed lncRNAs, such as MALAT1 and CDKN2B-AS1, and histone-modifying or chromatin-remodeling complexes has been implicated in transcriptional regulation, enabling the progression of different cancer types (Hanly et al., 2018). In this study, first, 36 HARGs were confirmed from previous research studies. Then, the RNA expression profile of these genes from the TCGA and GTEx datasets were collected, and 628 differentially expressed HARlncRNAs were obtained through co-expression and differential expression analysis. Univariate Cox regression, multivariate Cox regression, and the LASSO analyses were conducted step by step to establish a 14 HARlncRNAs risk signature, which was verified in the train, test, and entire set, respectively. The result presented that the risk score might be served as an independent prognostic biomarker for OC with an excellent predicting ability for survival. Importantly, the risk signature was further validated in the ICGC cohort.

It was well-known that cancer patients with HRD phenotype usually exhibit a high response to platinum compound chemotherapy (Pennington et al., 2014). Moreover, patients with HRD caused by different etiologies might be more suitable for the treatment with PARPis. In this study, the significantly higher HRD score and higher levels of HRD_LOH, HRD_ LST, and HRD_TAI were observed in the low-risk group than in the high-risk group, indicating that the low-risk group patients might benefit more from PARPis treatments. There existed different molecular features between the groups. The low-risk group showed the higher level of LOH_frac_altered and mutLoad_nonsilent, indicating that the low-risk group seemed to carry a higher mutation burden. DDR pathway deficiencies were demonstrated to be associated with mutation burden and mutational diversity, which might potentiate the response to immune therapies. In addition, the eCARD score was obviously higher in the low-risk group, which was used to investigate chemo-sensitivity in ovarian cancer patients from TCGA. The result might aid in the prediction for the response to platinum chemotherapy according to the risk score.

In OC, hundreds of lncRNAs were observed to be differentially expressed between OC and normal control tissues (Wang et al., 2016). Recently published reviews summarized some examples of differentially expressed lncRNAs associated with OC prognosis (Abildgaard et al., 2019; Wang et al., 2019). Given the increasing evidence, it could be considered that lncRNAs could not only serve as prognostic and predictive markers but also as highly specific therapeutic targets. As for the 14 HARlncRNAs, LEMD1-AS1 suppresses OC progression through sponging miR-183-5p and regulation of TP53 (Guo and Qin, 2020). Moreover, the prognosis implication of LEMD1-AS1 has been demonstrated in OC in the previous study (Zheng et al., 2020). AC011445.1, an lncRNA involved in immune and autophagy, was shown to be associated with OC patients’ clinical outcomes (Meng et al., 2020; Peng et al., 2022). However, the other HARlncRNAs have not been reported in OC up to date.

There were still some existing limitations in this research. It was necessary to further study the functions and molecular mechanisms of these 14 HARlncRNAs in combination with more experiments in OC; this may be helpful to find out HARlncRNAs that can be used as therapeutic targets. In addition, a larger sample size was needed to verify the accuracy of the risk signature and molecular subtypes in the future.

## Conclusion

To summarize, a risk signature composed of 14 HARlncRNAs was provided in OC. Significant differences in the survival rate and HRD status were observed between the high- and low-risk groups, which might provide the effective prediction for clinical outcomes and individual therapeutic strategies for patients with OC. Further lab experiments were needed to explore the molecular mechanisms of these 14 lncRNAs involved in histone acetylation *in vitro* and *in vivo*. In addition, a larger clinical sample size of OC was needed to recruit and validate the accuracy and efficacy of the 14-HARlncRNA signature.

## Data Availability

Publicly available datasets were analyzed in this study. These data can be found at https://xenabrowser.net.
